# Efficacy of Acupuncture in Post-partum With Diastasis Recti Abdominis: A Randomized Controlled Clinical Trial Study Protocol

**DOI:** 10.3389/fpubh.2021.722572

**Published:** 2021-12-13

**Authors:** Yan Liu, Ying Zhu, Liyuan Jiang, Chao Lu, Lijuan Xiao, Jiayu Chen, Ting Wang, Lujun Deng, Haida Zhang, Yingying Shi, Tingting Zheng, Min Feng, Tiantian Ye, Jing Wang

**Affiliations:** ^1^The Second Clinical Medical College, Zhejiang Chinese Medical University, Hangzhou, China; ^2^Zhejiang Chinese Medical University, Hangzhou, China; ^3^Department of Acupuncture and Rehabilitation, Hangzhou Hospital of Traditional Chinese Medicine, Hangzhou, China; ^4^Chun'an County Hospital of Traditional Chinese Medicine, Hangzhou, China; ^5^Dingqiao Hospital of Hangzhou Hospital of Traditional Chinese Medicine, Hangzhou, China; ^6^Department of Rehabilitation Medicine, The Sixth Affiliated Hospital of Sun Yat-sen University, Guangzhou, China; ^7^Department of Maternal Health Care, Maternity and Child Health Care Centers of Hechi, Hechi, China; ^8^Department of Rehabilitation, The Third Affiliated Hospital of Zhejiang Chinese Medical University, Hangzhou, China

**Keywords:** acupuncture, diastasis recti abdominis, post-partum, intrabdominis stimulation, randomized controlled trial

## Abstract

**Background:** Diastasis rectus abdominis (DRA) is one of the common complications during pregnancy and post-partum, which has psychological and physical effects on post-partum women. Acupuncture, a worldwide alternative therapy, has attracted wide attention in preventing and treating diseases related to pregnancy and childbirth. This study aims to evaluate the efficacy of acupuncture combined with physical training in treating post-partum rectus muscle dissociation.

**Methods:** This is a randomized, controlled trial of DRA in post-partum conducted at Hangzhou Hospital of Traditional Chinese Medicine Affiliated with Zhejiang University of Chinese Medicine. The primary purpose is to evaluate the effectiveness of acupuncture and physical training on DRA in post-partum women. The study will be conducted from March 2022 to March 2023. The acupuncture group received acupuncture and physical training (*n* = 48), the sham acupuncture group received sham acupuncture and physical training (*n* = 48), and the physical training group received physical training (*n* = 48). These experiments perform once/day, five times a week for 2 weeks, followed up for half a year after the end of the course of treatment. Our tests perform a course of treatment, which includes a total of 10 consecutive treatments. Furthermore, the patient will be followed up for half a year after the treatment. Primary and secondary indicators, including inter recti distance (IRD), linea alba (LA) tension, the MOS item short-form health survey (SF-36), short-form McGill pain questionnaire-2 (SF-MPQ-2), body mass index (BMI), waist-to-hip ratio (WHR), leeds dyspepsia questionnaire (LDQ), menstrual distress questionnaire (MDQ), 10 items of edinburgh post-natal depression scale (EPDS-10), the modified body self-image scale (MBIS), international consultation incontinence questionnaire short-form (ICIQ-SF) and hernia-related quality-of-life survey (HerQles), which will be evaluated before and after treatment and half a year after treatment. Adverse events and side effects during each treatment will be collected and recorded.

**Discussion:** There is evidence that acupuncture and physical training can treat DRA in post-partum. In this study, we evaluate the effectiveness of acupuncture in post-partum with DRA.

## Highlights

- Between 35 and 70% of post-partum rectus muscle dissections fail to recover without any treatment or exercise.
- This paper presents a treatment method for post-partum rectus abdominal separation.
- Acupuncture for post-partum rectus separation as a technique of internal rectus stimulation.

## Background

Diastasis recti abdominis is a condition in which both rectus abdominal muscles disintegrate to the sides, accompanied by the extension of the linea alba tissue and bulging of the abdominal wall (1). 30–70% of pregnant women develop DRA during pregnancy (2). Between 35 and 70 percent of pregnant women do not recover after giving birth without treatment or exercise (3). In addition, 39% to 45% of women still have DRA at 6 months post-partum, and the incidence of DRA at 1 year post-partum is 23–32% (1). DRA has both psychological and physical effects on post-partum women. Women with DRA primarily receive the application of support band and abdominal band during pregnancy and post-partum (4), electrical stimulation, surgical repair (5), and physical training (6). There is a lack of unified and effective treatment, and there are few studies on the efficacy and safety of DRA physical training (5, 7). As a worldwide alternative therapy, acupuncture has received wide attention in preventing and treating diseases related to pregnancy and childbirth.

Acupuncture combined with physical training can significantly improve tissue excitability, which improves blood circulation and systolic muscle function (8) and adjust the mechanical balance of the post-partum abdominal muscle group. In abdominal physical training, the increase of abdominal pressure makes the pelvic floor vulnerable to adverse effects and causes or exacerbates pelvic floor dysfunction. Acupuncture can well cause the secondary synergism of pelvic floor muscles by activating the transverse abdomens (9, 10).

However, the long-term efficacy is still unclear, and there is a lack of solid objective evidence. To date, no RCT studies the impact of acupuncture on DRA or evaluates the standardized application of DRA by acupuncture. This study comprehensively evaluates the effectiveness and safety of acupuncture in the treatment of post-partum DRA, the effective, and safety of DRA physical training. It provides a reference for clinical treatment of post-partum DRA.

### Hypothesis

The hypothesis is that intervention with acupuncture is better than physical training in these outcomes of the inter recti distance (IRD) of DRA. We also expect to prove that acupuncture can be an ideal safe and conservative alternative for patients with DRA who fail in conventional physiotherapy.

## Methods

This study protocol was developed under the Recommendations for Interventional Trials 2013 Statement (SPIRIT 2013) (11) (Table 1) and the Consolidated Reporting Trials statement (CONSORT 2010) when applicable (12).

**Table 1:** SPIRIT 2013 checklist: recommended items to address in a clinical trial protocol and related documents^*^.

**Section/item**	**Item no**.	**Description**	**Yes/No**
**Administrative information**			
Title	1	Descriptive title identifying the study design, population, interventions, and, if applicable, trial acronym	Yes
Trial registration	2a	Trial identifier and registry name. If not yet registered, name of intended registry	Yes
	2b	All items from the World Health Organization Trial Registration Data Set	Yes
Protocol version	3	Date and version identifier	Yes
Funding	4	Sources and types of financial, material, and other support	Yes
Roles and responsibilities	5a	Names, affiliations, and roles of protocol contributors	Yes
	5b	Name and contact information for the trial sponsor	Yes
	5c	Role of study sponsor and funders, if any, in study design; collection, management, analysis, and interpretation of data; writing of the report; and the decision to submit the report for publication, including whether they will have ultimate authority over any of these activities	Yes
	5d	Composition, roles, and responsibilities of the coordinating center, steering committee, endpoint adjudication committee, data management team, and other individuals or groups overseeing the trial, if applicable (see Item 21a for data monitoring committee)	Yes
**Introduction**			
Background and rationale	6a	Description of research question and justification for undertaking the trial, including summary of relevant studies (published and unpublished) examining benefits and harms for each intervention	Yes
	6b	Explanation for choice of comparators	Yes
Objectives	7	Specific objectives or hypotheses	Yes
Trial design	8	Description of trial design including type of trial (e.g., parallel group, crossover, factorial, single group), allocation ratio, and framework (eg, superiority, equivalence, non-inferiority, exploratory)	Yes
**Methods: participants, interventions, and outcomes**			
Study setting	9	Description of study settings (eg, community clinic, academic hospital) and list of countries where data will be collected. Reference to where list of study sites can be obtained	Yes
Eligibility criteria	10	Inclusion and exclusion criteria for participants. If applicable, eligibility criteria for study centers and individuals who will perform the interventions (eg, surgeons, psychotherapists)	Yes
Interventions	11a	Interventions for each group with sufficient detail to allow replication, including how and when they will be administered	Yes
	11b	Criteria for discontinuing or modifying allocated interventions for a given trial participant (eg, drug dose change in response to harms, participant request, or improving/worsening disease)	Yes
	11c	Strategies to improve adherence to intervention protocols, and any procedures for monitoring adherence (eg, drug tablet return, laboratory tests)	Yes
	11d	Relevant concomitant care and interventions that are permitted or prohibited during the trial	Yes
Outcomes	12	Primary, secondary, and other outcomes, including the specific measurement variable (eg, systolic blood pressure), analysis metric (eg, change from baseline, final value, time to event), method of aggregation (eg, median, proportion), and time point for each outcome. Explanation of the clinical relevance of chosen efficacy and harm outcomes is strongly recommended	Yes
Participant timeline	13	Time schedule of enrolment, interventions (including any run-ins and washouts), assessments, and visits for participants. A schematic diagram is highly recommended (see Figure)	Yes
Sample size	14	Estimated number of participants needed to achieve study objectives and how it was determined, including clinical and statistical assumptions supporting any sample size calculations	Yes
Recruitment	15	Strategies for achieving adequate participant enrolment to reach target sample size	Yes
**Methods: assignment of interventions (for controlled trials)**			
Allocation:			Yes
Sequence generation	16a	Method of generating the allocation sequence (eg, computer-generated random numbers), and list of any factors for stratification. To reduce predictability of a random sequence, details of any planned restriction (eg, blocking) should be provided in a separate document that is unavailable to those who enroll participants or assign interventions	Yes
Allocation concealment mechanism	16b	Mechanism of implementing the allocation sequence (eg, central telephone; sequentially numbered, opaque, sealed envelopes), describing any steps to conceal the sequence until interventions are assigned	Yes
Implementation	16c	Who will generate the allocation sequence, who will enroll participants, and who will assign participants to interventions	Yes
Blinding (masking)	17a	Who will be blinded after assignment to interventions (eg, trial participants, care providers, outcome assessors, data analysts), and how	Yes
	17b	If blinded, circumstances under which unblinding is permissible, and procedure for revealing a participant's allocated intervention during the trial	Yes
**Methods: data collection, management, and analysis**			
Data collection methods	18a	Plans for assessment and collection of outcome, baseline, and other trial data, including any related processes to promote data quality (eg, duplicate measurements, training of assessors) and a description of study instruments (eg, questionnaires, laboratory tests) along with their reliability and validity, if known. Reference to where data collection forms can be found, if not in the protocol	Yes
	18b	Plans to promote participant retention and complete follow-up, including list of any outcome data to be collected for participants who discontinue or deviate from intervention protocols	Yes
Data management	19	Plans for data entry, coding, security, and storage, including any related processes to promote data quality (eg, double data entry; range checks for data values). Reference to where details of data management procedures can be found, if not in the protocol	Yes
Statistical methods	20a	Statistical methods for analyzing primary and secondary outcomes. Reference to where other details of the statistical analysis plan can be found, if not in the protocol	Yes
	20b	Methods for any additional analyses (eg, subgroup and adjusted analyses)	Yes
	20c	Definition of analysis population relating to protocol non-adherence (eg, as randomized analysis), and any statistical methods to handle missing data (eg, multiple imputation)	Yes
**Methods: monitoring**			
Data monitoring	21a	Composition of data monitoring committee (DMC); summary of its role and reporting structure; statement of whether it is independent from the sponsor and competing interests; and reference to where further details about its charter can be found, if not in the protocol. Alternatively, an explanation of why a DMC is not needed	Yes
	21b	Description of any interim analyses and stopping guidelines, including who will have access to these interim results and make the final decision to terminate the trial	Yes
Harms	22	Plans for collecting, assessing, reporting, and managing solicited and spontaneously reported adverse events and other unintended effects of trial interventions or trial conduct	Yes
Auditing	23	Frequency and procedures for auditing trial conduct, if any, and whether the process will be independent from investigators and the sponsor	Yes
**Ethics and dissemination**			
Research ethics approval	24	Plans for seeking research ethics committee/institutional review board (REC/IRB) approval	Yes
Protocol amendments	25	Plans for communicating important protocol modifications (eg, changes to eligibility criteria, outcomes, analyses) to relevant parties (eg, investigators, REC/IRBs, trial participants, trial registries, journals, regulators)	Yes
Consent or assent	26a	Who will obtain informed consent or assent from potential trial participants or authorized surrogates, and how (see Item 32)	Yes
	26b	Additional consent provisions for collection and use of participant data and biological specimens in ancillary studies, if applicable	Yes
Confidentiality	27	How personal information about potential and enrolled participants will be collected, shared, and maintained in order to protect confidentiality before, during, and after the trial	Yes
Declaration of interests	28	Financial and other competing interests for principal investigators for the overall trial and each study site	Yes
Access to data	29	Statement of who will have access to the final trial dataset, and disclosure of contractual agreements that limit such access for investigators	Yes
Ancillary and post-trial care	30	Provisions, if any, for ancillary and post-trial care, and for compensation to those who suffer harm from trial participation	Yes
Dissemination policy	31a	Plans for investigators and sponsor to communicate trial results to participants, healthcare professionals, the public, and other relevant groups (eg, via publication, reporting in results databases, or other data sharing arrangements), including any publication restrictions	Yes
	31b	Authorship eligibility guidelines and any intended use of professional writers	Yes
	31c	Plans, if any, for granting public access to the full protocol, participant-level dataset, and statistical code	Yes
**Appendices**			
Informed consent materials	32	Model consent form and other related documentation given to participants and authorized surrogates	Yes
Biological specimens	33	Plans for collection, laboratory evaluation, and storage of biological specimens for genetic or molecular analysis in the current trial and for future use in ancillary studies, if applicable	Not applicable

* *It is strongly recommended that this checklist be read in conjunction with the SPIRIT 2013 Explanation and Elaboration for important clarification on the items. Amendments to the protocol should be tracked and dated. The SPIRIT checklist is copyrighted by the SPIRIT Group under the Creative Commons “Attribution-NonCommercial-NoDerivs 3.0 Unported” license*.

### Study Design and Setting

**Figure 1:**
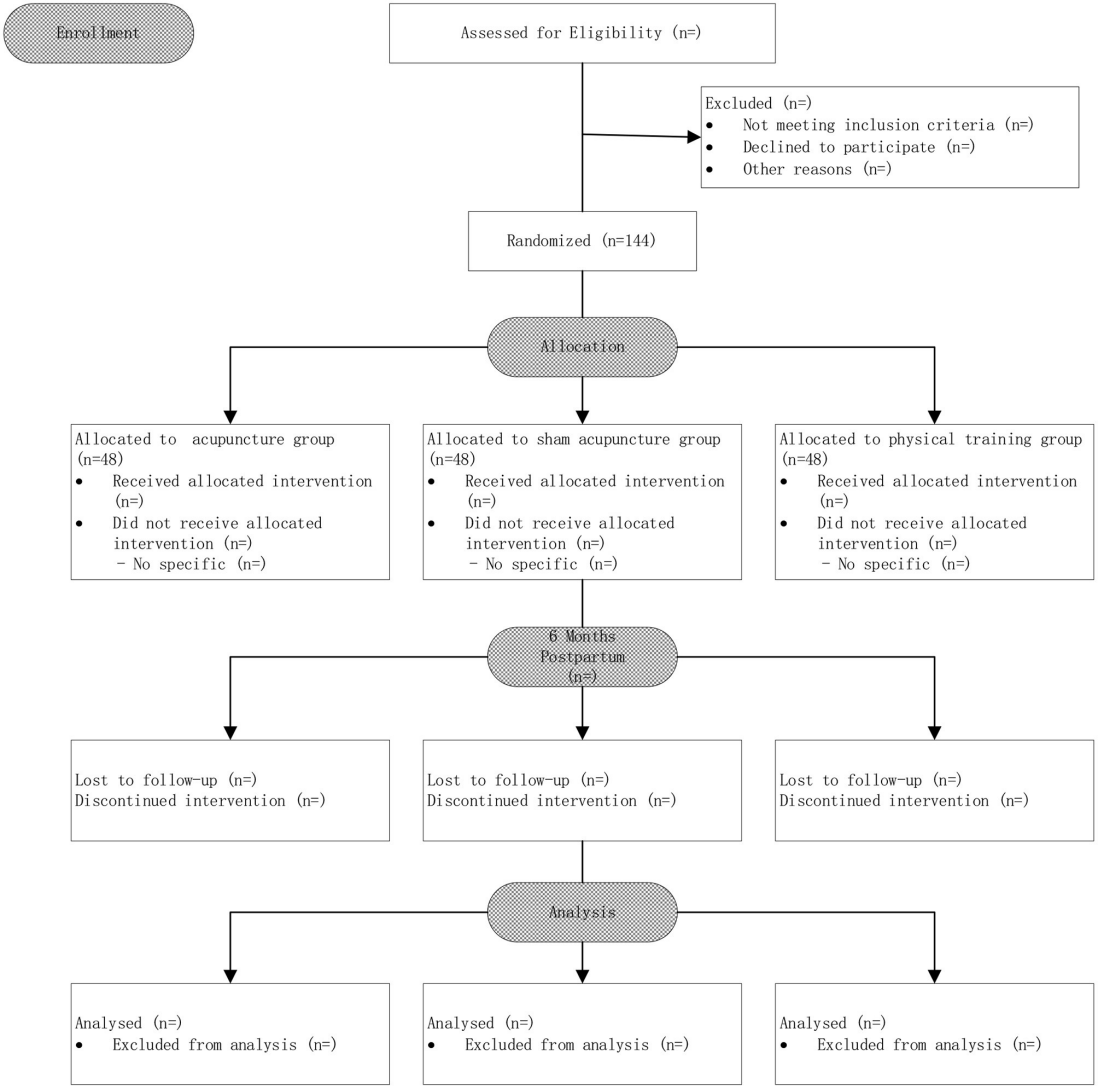
Flow chart of the study design.

This randomized, controlled clinical trial is carried out at the Hangzhou Hospital of Traditional Chinese Medicine Affiliated to Zhejiang Chinese Medical University, Hangzhou, China (Figure 1).

### Sample Size

According to previous similar reports (13), the average value of IRD in the physical training group was 2.09 after treatment, and the average value of IRD in the acupuncture and physical training group was expected to be 1.43 after treatment in this study. Three groups were set up in this study. The test level was α = 0.05, the test efficiency was 1–*β* = 0.90, and the two-sided test was conducted. The sample size was estimated by PASS15.0 software (13), effectiveness size is 0.313249. Considering 2-sided *P*-values to be deemed statistically significant at *P* < 0 0.05 and a power of 90%, 43 participants would be required per group (nQuery Advisor, version 4.0; Statistical Solutions). Estimating that 10% of patients might be lost to follow-up, we planned to enroll a total of 144 participants, with 48 in each group.

### Participants and Recruitment

The study was conducted from March 2022 to March 2023. The acupuncture group received acupuncture and physical training (*n* = 48), the sham acupuncture group received sham acupuncture and physical training (*n* = 48), and the physical training group received physical training (*n* = 48).

Participants were recruited from inclusion criteria:

(1) A female aged 18–45 years with no previous history of pathological rectus abdominal dissection;
(2) Vaginal delivery, 42 days to 1 year post-partum;
(3) DRA diagnostic criteria:
  - The patient should keep in the supine position, with knees bending about 90°, the whole body relaxed, and the soles of the feet flat. The examiner shall put the abdomen of one hand finger on the umbilical level of the patient;
  - The patient is told to breathe through the abdomen and to lift one's head and shoulders slightly off the bed;

Measuring the width of the finger inserted by the examiner, DRA is diagnosed when IRD ≥ 2 cm (14–16).

(4) No cognitive barriers, able to understand and communicate correctly;
(5) Have not received treatment for DRA in other hospitals or institutions;
(6) Those who sign the informed consent cooperate with the treatment and can adhere to the completion of all treatment as planned.

Patients who meet the above six criteria can be included in this study.

The subjects studied for DRA in post-partum women should follow the following exclusion criteria:

(1) One is suspected or diagnosed with severe spinal lesions (such as spinal fractures, metastases, inflammatory or infectious diseases, cauda equina syndrome/widespread neurological disease) and neurological injury;
(2) One has motor contraindications or severe infectious diseases such as fractures, severe heart disease, hypertension, cancer;
(3) Any plans for surgery soon;
(4) History of cesarean section;
(5) Abdominal operation history;
(6) Any of the above shall be excluded.

### Randomization and Binding

A total of 144 eligible patients were recruited and randomly assigned at a 1:1:1 ratio to receive acupuncture treatment or sham acupuncture treatment or be placed on a physical training. Central randomization, using an online or messaging system, was performed by the Brightech Magnsoft Data Services. The randomization sequence was generated in blocks of varying sizes and stratified by centers.

The acupuncture and sham acupuncture groups were blinded, while those in the physical training group were not. All clinical examinations were performed by Haida Zhang and Lujun Deng physiotherapists blinded to data collected through the questionnaire. Due to iodophor disinfection traces in the abdomen after electroacupuncture, the therapists knew the groups, so Yingying Shi performed electroacupuncture treatment after rehabilitation exercise first. All the therapists did not know the purpose of the experiment.

The acupuncture and sham acupuncture groups were blinded, while those in the physical training group were not. All clinical examinations were performed by Haida Zhang and Lujun Deng, two physiotherapists blinded to data collected. Due to iodophor disinfection traces in the abdomen after electroacupuncture, the therapists knew the groups. So Yingying Shi performed electroacupuncture treatment after rehabilitation exercise first. Outcome assessors, data collectors, and statisticians did not know the purpose of the experiment and were blinded to the treatment allocation.

### Intervention

**Figure 2:**
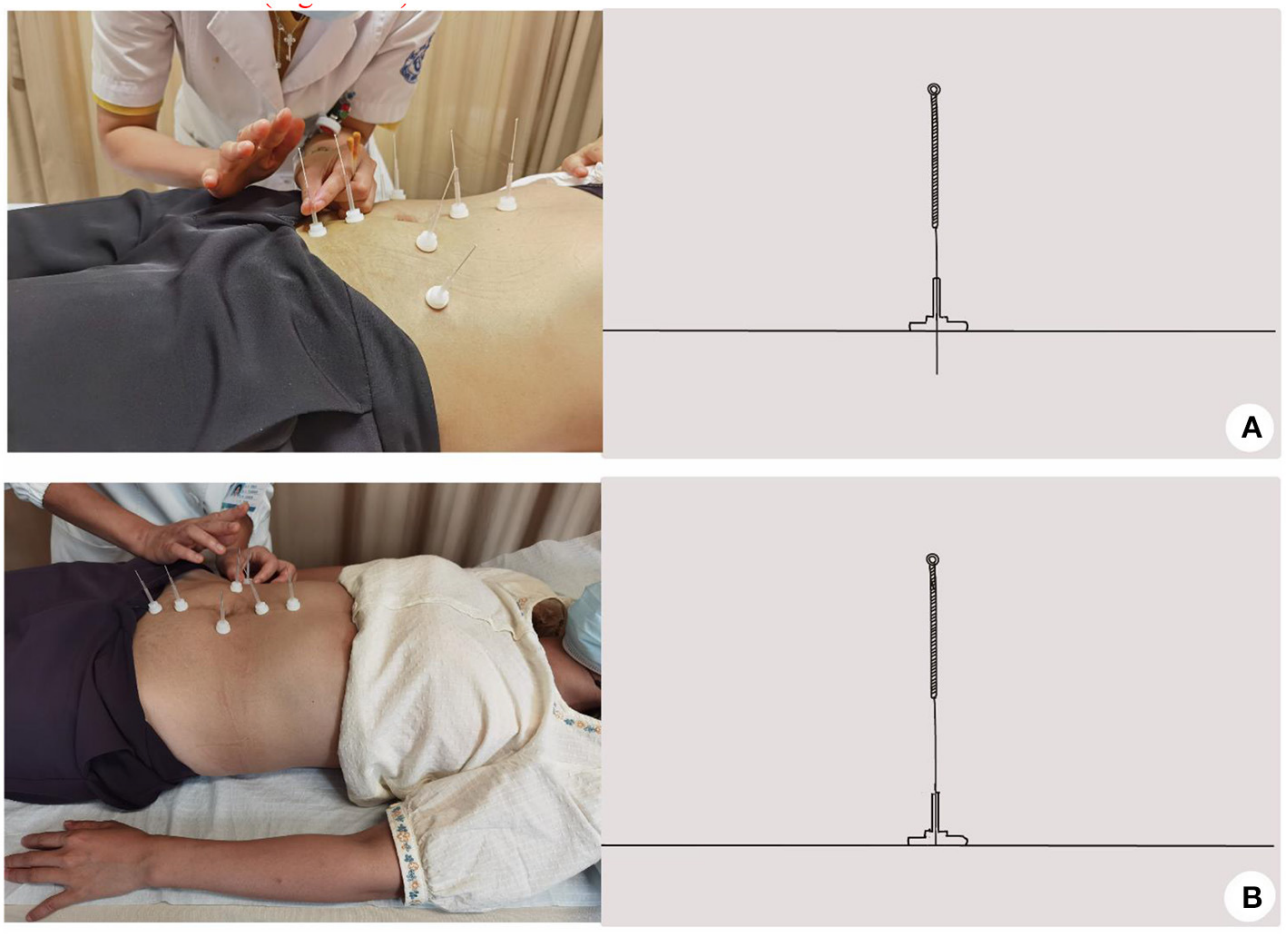
The schematic diagram of acupuncture and sham acupuncture group. **(A)** Acupuncture group; **(B)** Sham acupuncture group.

**Figure 3:**
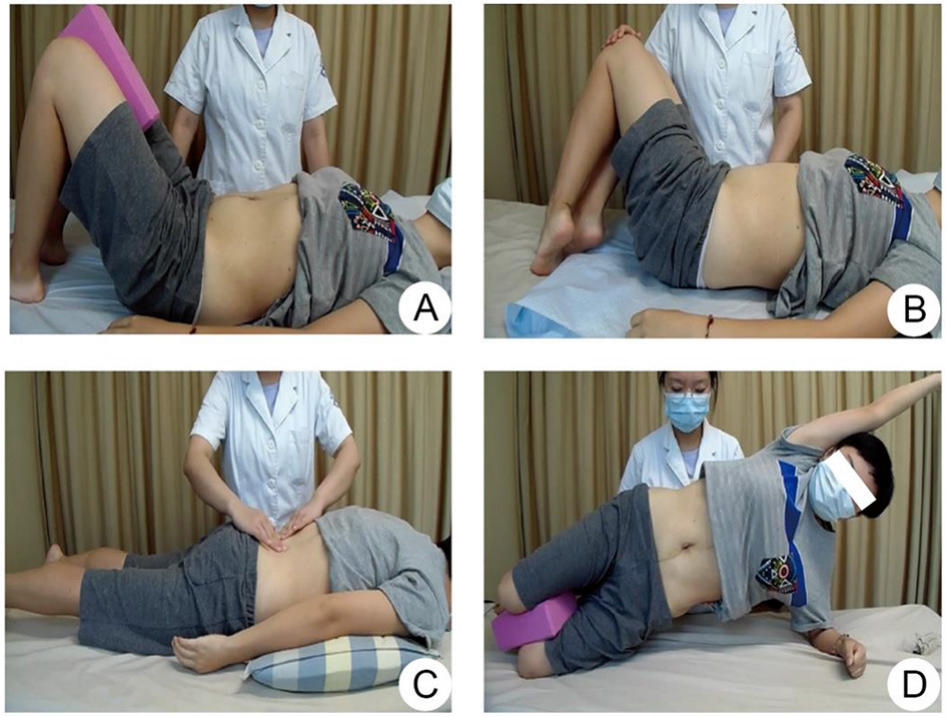
The flow chart of physical training group. **(A)** Abdominal breathing; **(B)** Left and right-side leg rotation; **(C)** Stretch legs after stomach retraction; **(D)** Ventral flat training.

1. Acupuncture group: the patient was placed in the supine position, exposing the abdomen, and acupoints RN12 (Zhongwan), RN10 (Xiawan), ST25 (Tianshu), GB26 (Dai Mai), RN6 (Qi Hai), and RN4 (Guanyuan) were selected. The selection of acupoints was based on the previous literature and clinical experience of DRA. The skin at the acupoints was routinely disinfected, and the disposable sterile acupuncture needles were used for vertical acupuncture of 25–40 mm, 30 min once/day, five times a week for 2 weeks (Figure 2A).
2. Sham acupuncture group: non-penetrating sham acupuncture is performed on the same points as the treatment group on the abdomen. Streitberger placebo needles with blunt tips are used. When they are fixed on the skin through plastic rings, patients will feel a pricking sensation, simulating a skin puncture. However, instead of penetrating the skin, the needles retract up into the shaft when pressed against the skin. We will formulate and follow standardize step-by-step instructions and operations to use the same rituals in the acupuncture and sham acupuncture groups as far as possible, 30 min once/day, five times a week for 2 weeks (Figure 2B).
3. Physical training group: (1) Abdominal breathing: supine position, lower limb hip, and knee flexion, foam bricks are clamped between the legs. the abdomen will be humped while inhaling and is forced to the navel while exhaling. Abdominal muscles and pelvic floor muscles are forced to contract at the same time, ten times a group for three groups. (2) Left and right-side leg rotation: buckling, supine, legs down to the right. inhale, abdominal bulge, expiratory when abdomen muscle contraction, to, make double leg down to the left (power is core abdominal muscles, legs, not too much), the therapist skill on the right side of the internal, external oblique muscle in patients with muscle contraction, the muscle belly line direction, the other patients on your knees. Apply some resistance according to the patient's strength of the force. Do it alternately, 10 times on each side. (3) Stretch legs after stomach retraction: Patients prone position, the abdominal pad is a soft pillow, inhale, abdominal bulge, expiratory when abdomen muscle contraction (imagine navel carried from pillow side), in turn, the waist, on the left side of the hips, on the left side of the thigh, to raise the left lower limb, the core in the waist, leg without over pressure, and avoid the contralateral compensatory, therapists place the fourth finger near the lumbar spine feeling the relaxation from torn muscle contraction. Alternating left and right, do 10 reps on each side. (4) Ventral flat training: the patient is in the right described position, elbow flexion support on the bed surface, hip extension, calf flexion and thigh 90 degrees, abdominal uplift when inhaling, abdominal muscles when exhaling, upper body, buttocks and left lower limb straight lift off the bed surface, left upper limb lift, upper arm against the head, ear sticking, maintain relaxation after 10 s. Core in the abdomen, other parts of the body cannot apply excessive force. Alternating left and right, do 10 reps on each side (Figure 3).

The trial must end when the following happened: (1) When severe adverse reactions occur on the patient during the trial; (2) When serious complications or deteriorations happen; (3) When the patient is asked to withdraw from the clinical study; (4) When the patient does not cooperate or accept any treatment with the treatment-personnel repeatedly explaining ineffective.

We gather the patients into a WeChat chatting group to answer their questions anytime to prevent the above situations. We try to cooperate and share any knowledge of DRA with our patients to contact them more fluently and smoothly. Furthermore, when the patient gets any other problems, we will have the treatment and record the changes in the patient's condition. During the trial, any other treatment on DRA is not allowed in case of being interfered with. If one happened, the patient must withdraw from the study.

### Primary Outcome

We consider the IRD as the primary outcome of DRA treatment (7). The same ultrasonic doctor will measure and evaluate before and after the treatment and after a 6-month follow-up.

### Secondary Outcomes

1. LA tension evaluation: IRD, continuous midpoint of umbilical cord and knife-edge, level of the umbilical cord, can be measured by ultrasound. Elastography images can be graded using a modified five-point method (17). The scoring criteria were as follows:1 point indicated that most of the lesions or lesions were green; 2 points are red and green areas with <20%; 3 points means the red area is >20% but <50%; 4 points are areas with or without green that are more than 50% red but <80% or more than 80%. Measurements and assessments were made before and after treatment and after 6 months of follow-up.
2. SF-36: It includes physical function, physical pain, general health status, energy, social function, emotional function, mental health, and indicators of health changes in the past year (18). It can reflect a comprehensive summary of the quality of life of the respondents. The higher the score, the healthier you are. Cronbach's α coefficient was 0.838, and Spearman-Brown coefficient was 0.828, indicating good reliability and validity. Measurement and evaluation will be performed before and after the 6-month follow-up.
3. SF-MPQ-2: This is a systemic pain assessment for patients with low back pain. The following quantitative/semi-quantitative data can be obtained from this scale: (1) PRI: The total score of all selected words. (2) NCW: VAS evaluation in the chart. (3) PPI: 6-degree NRS was used to evaluate the total pain intensity. Cronbach's α coefficient is more significant than 0.8, indicating reasonable confidence and validity. Measurements and assessments were made before treatment and after 6 months of follow-up.
4. BMI: The index is used to assess whether a person is thin or overweight. The ratio of being too small (<18.5 for Asian adults) or too large (≥24 for Asian adults) represents an increased incidence of certain diseases and reflects human health status. Measurements and assessments were made before and after treatment and after six months of follow-up.
5. WHR: It is an essential indicator of central obesity and female attractiveness. Measurements and assessments were made before and after treatment and after 6 months of follow-up.

### Other Observation Items

1. LDQ: The questionnaire is a dimensional-symptom-specific scale tool for assessing disease conditions. The Cronbach's α coefficient of the table is 0.908, and the Cronbach's α coefficient of each dimension is 0.448–0.990, indicating good internal consistency and high measurement reliability (19). Measurements and assessments were made before and after treatment and after 6 months of follow-up.
2. MDQ: To assess the status of the menstrual disease. The overall Cronbach's α coefficient was 0.937 (*P* < 0.001), and the Cronbach's α coefficients of each dimension ranged from 0.652 to 0.862 (20). Measurements and assessments were made before and after treatment and after 6 months of follow-up.
3. EPDS-10: The revised EPDS has a confidence of 0.76, content validity of 0.93, and a recommended critical value of 9.5 points (21). The measurement and evaluation will be taken before and after the treatment and after a 6-month follow-up.
4. MBIS: MBIs have been shown to work via changes in specific aspects of psychopathology, such as cognitive biases, affective dysregulation, and interpersonal effectiveness (22). The measurement and evaluation will be taken before and after the treatment and after a 6-month follow-up.
5. ICIQ-SF: International Association for Urine Control Questionnaire. The Chinese version of Cronbach's α coefficient indicates an internal consistency of 0.72–0.8 (22). After the inspection. Measurements and assessments were made before and after treatment and after 6 months of follow-up.

### Adverse Events Reporting and Safety Monitoring

Safety: Adverse events (AE), side effectiveness (SE) such as falls, joint sprains will be recorded during the evaluation.

Compliance: Incomplete information and number of shedding for the two selected cases were recorded during and after the evaluation.

Acupuncture and exercise training were used as intervention items in this study. Before participating in the study, each subject underwent clinical diagnosis and contraindications, and detailed records were made during the treatment process. DURING THE EXPERIMENT, any AE, SE (fall, joint sprain, bleeding, post-stitch, needle blocking, etc.) will be recorded in detail and reported to the project leader. Subjects with adverse events will be treated in the hospital where the project is being implemented, and the project team will bear the treatment and examination costs.

### Data Analysis

Based on Preliminary experiment results, we estimated that each of the three clinical researchers involved in the trial would recruit around 28 patients in 3 months. In order to retain most of the patients during follow-up, we gather the patients into a WeChat chatting group to answer their questions anytime. Moreover, actively cooperate with their appointment time. We expect the loss to follow-up to be below 10%.

Baseline characteristics and clinical outcomes are described based on the intention-to-treat population (*n* = 144). Continuous variables are presented as mean (SD), and categorical variables are described as number and percentage.

The principal analysis for primary and secondary outcomes will be a generalized linear model (GLM) including the intervention arm, the dependent variable's baseline score, and other variables imbalanced at baseline as covariates. The dependent variable will be the post-intervention score. The GLM does not need a normal distribution in the dependent variable. However, the distribution of the variables will be analyzed using de Shapiro- Wilk test to perform an adequate descriptive analysis. The choice of the link function will depend on the distribution of the variable.

Other variables will be added as confounders in the model as secondary sensitivity analyses. We will first fit separate models, including each confounder, one at a time. Those variables whose inclusion in the model changes the estimates' treatment effectiveness by at least 10% will be considered as confounders. As suggested in the CONSORT statement, decisions about confounders will not be based on *P*-value.

Missing data of withdrawn participants will be accommodated with multiple imputation procedures in Stata 25.0 software (Stata Corporation). All *P*-values were from 2-sided tests, and results were deemed statistically significant at *P* < 0.05.

## Discussion

There is evidence that physical training can improve DRA (13, 23). To date, however, previous research focuses on how to reduce IRD for DRA treatment (24) but ignores its safety and effectiveness on physiological, psychological, and other systemic regulation. In the process of DRA exercise rehabilitation, individual differences in proprioception of abdominal wall and pelvic floor and cooperative motor control ability should be considered, and patient education and self-perception ability training should be paid attention to. At present, there are no published data on the effectiveness of acupuncture in post-partum with Diastasis Recti Abdominis by A Randomized Controlled Clinical Trial Study.

Increasing adherence to self-care is a challenge in diseases such as DRA. An acupuncture-based intervention to replace single physical training may improve DRA in the future. This study aims to evaluate the effectiveness and safety of acupuncture in post-partum with Diastasis Recti Abdominis to bring the gospel to most DRA patients.

## Limitation

First, the extrapolation of the research conclusions of RCT to clinical practice is faced with challenges, e.g. limited sample size. Besides, it is difficult to implement traditional RCT during patient follow-up.

## Ethics Statement

The studies involving human participants were reviewed and approved by the Ethics Committee of Hangzhou Hospital of TCM. The patients/participants provided their written informed consent to participate in this study.

## Author Contributions

YL: data analyses, figure preparation, and manuscript preparation. YZ: questionnaire evaluation and manual data measurement before treatment. LJ: responsible for the design of randomization, project funding, and study initiation. CL: ultrasound evaluation. LX: responsible for the design of randomization. JC, TW, and TY: responsible for data entry. LD and HZ: recovery treatment. YS: acupuncture treatment. TZ, MF, and JW: responsible for guidance and statistics. All authors approved the final version of the manuscript.

## Funding

This RCT was funded by Zhejiang Traditional Chinese Medicine Science and Technology Plan Project (Project Number: 2021ZQ065).

## Conflict of Interest

The authors declare that the research was conducted in the absence of any commercial or financial relationships that could be construed as a potential conflict of interest.

## Publisher's Note

All claims expressed in this article are solely those of the authors and do not necessarily represent those of their affiliated organizations, or those of the publisher, the editors and the reviewers. Any product that may be evaluated in this article, or claim that may be made by its manufacturer, is not guaranteed or endorsed by the publisher.

## Abbreviations

AE: Adverse Event
BMI: Body Mass Index
DRA: Diastasis Recti Abdominis
OR: Odds Ratio
PASS: Power Analysis and Sample Size
IRD: Inter Recti Distance
PRI: Pain Rating Index
NWC: Number of Words Chosen
PPI: Present Pain Intensity
ICC: Intraclass Correlation Coefficients
WHR: Waist-to-Hip Ratio
LDQ: Leeds Dyspepsia Questionnaire
MDQ: Menstrual Distress Questionnaire
MI: Multiple Imputation
PMM: Pattern Mixture Model
SF-36: The MOS Item Short Form Health Survey
LA: Linea Alba
SF-MPQ-2: Short-Form McGill Pain Questionnaire-2
EPDS-10: 10 Items of Edinburgh Post-natal Depression Scale
MBIS: The Modified Body Self-Image Scale
ICIQ-SF: International Consultation Incontinence Questionnaire Short-Form
HerQles: Hernia-Related Quality-of-Life Survey
SE: Side Event
GLM: Generalized linear model.
